# Targeting Chemosensory Ion Channels in Peripheral Swallowing-Related Regions for the Management of Oropharyngeal Dysphagia

**DOI:** 10.3390/ijms21176214

**Published:** 2020-08-27

**Authors:** Mohammad Zakir Hossain, Hiroshi Ando, Shumpei Unno, Junichi Kitagawa

**Affiliations:** 1 Department of Oral Physiology, School of Dentistry, Matsumoto Dental University, 1780 Gobara Hirooka, Shiojiri, Nagano 399-0781, Japan; shumpei.unno@mdu.ac.jp; 2 Department of Biology, School of Dentistry, Matsumoto Dental University, 1780 Gobara, Hirooka, Shiojiri, Nagano 399-0781, Japan; hiroshi.ando@mdu.ac.jp

**Keywords:** oropharyngeal dysphagia, chemosensory ion channels, peripheral chemical neurostimulation strategy, neurophysiological mechanisms, molecular mechanisms

## Abstract

Oropharyngeal dysphagia, or difficulty in swallowing, is a major health problem that can lead to serious complications, such as pulmonary aspiration, malnutrition, dehydration, and pneumonia. The current clinical management of oropharyngeal dysphagia mainly focuses on compensatory strategies and swallowing exercises/maneuvers; however, studies have suggested their limited effectiveness for recovering swallowing physiology and for promoting neuroplasticity in swallowing-related neuronal networks. Several new and innovative strategies based on neurostimulation in peripheral and cortical swallowing-related regions have been investigated, and appear promising for the management of oropharyngeal dysphagia. The peripheral chemical neurostimulation strategy is one of the innovative strategies, and targets chemosensory ion channels expressed in peripheral swallowing-related regions. A considerable number of animal and human studies, including randomized clinical trials in patients with oropharyngeal dysphagia, have reported improvements in the efficacy, safety, and physiology of swallowing using this strategy. There is also evidence that neuroplasticity is promoted in swallowing-related neuronal networks with this strategy. The targeting of chemosensory ion channels in peripheral swallowing-related regions may therefore be a promising pharmacological treatment strategy for the management of oropharyngeal dysphagia. In this review, we focus on this strategy, including its possible neurophysiological and molecular mechanisms.

## 1. Introduction

Swallowing is a physiological process that transports ingested materials (e.g., foods and liquids) and saliva from the oral cavity into the stomach [1,2,3,4,5]. It is a highly integrated and complex sensorimotor process that has both volitional and reflexive components [1,2,3,4,5]. Depending on the anatomical locations of the ingested material, the process of swallowing can been divided into three phases: Oral, pharyngeal, and esophageal [1,2,3,4,5]. The oral phase is volitional and includes the process of taking materials into the oral cavity, and the preparation by chewing, or mastication, of a bolus of suitable size and consistency to be swallowed [1,2,3,4,5]. In the pharyngeal phase, the bolus is transferred into the esophagus by a reflex mechanism known as the swallowing reflex, which also prevents the bolus from entering the respiratory tract. During this reflex, the epiglottis swings down to cover the laryngeal vestibule and the laryngeal opening is closed off by the vocal cords and arytenoids, leading to a sealing of the airway [1,2,3,4,5]. The laryngeal vestibule closes to prevent the entry of the bolus into the trachea. The hyoid bone and larynx then move upward and forward, and the upper esophageal sphincter is elevated. Next, the upper esophageal sphincter opens, and the bolus moves to the esophagus. During the esophageal phase, the bolus and liquid are transported into the stomach with the aid of peristaltic contraction and gravity [1,2,3,4,5].

Difficulties in the process of swallowing are termed dysphagia. Swallowing difficulties often lead to severe complications, such as pulmonary aspiration, malnutrition, dehydration, and pneumonia, which have high mortality rates [6,7,8,9,10,11,12]. Generally, dysphagia is divided into oropharyngeal and esophageal subtypes based on the location of the swallowing difficulty [13,14,15]. In oropharyngeal dysphagia, difficulty arises when transporting the food bolus or liquid from the oral cavity to the esophagus, while in esophageal dysphagia, the impedance occurs in the esophagus itself [13,14,15]. Oropharyngeal dysphagia is more prevalent and more severe than esophageal dysphagia [16]. In oropharyngeal dysphagia, patients have difficulties with evoking swallowing. Triggering of the swallow is often delayed, leading to impaired safety of swallowing. If the swallow response is not evoked at the correct time, the airways may remain open during swallowing. This can allow the entry of food particles or liquids into the laryngeal vestibule above the vocal folds (termed penetration,) or even deep into the airway below the vocal folds (termed aspiration), and may lead to aspiration pneumonia [17,18]. Airway penetration and aspiration are caused by a delayed laryngeal vestibule closure time and slow hyoid motion [6,19]. Impaired safety of swallowing with bolus penetration occurs in more than half of all patients with oropharyngeal dysphagia, and approximately 20–25% of these patients present aspiration into the airway [6,20,21]. The inability to swallow efficiently can also lead to the presence of bolus residues in the oropharyngeal region (termed oropharyngeal residues), which causes the sensation of having food stuck in the oral cavity or throat regions [22,23]. Oropharyngeal residues occur because of weak bolus propulsion forces and impaired pharyngeal clearance [6,19]. There are many causes of oropharyngeal dysphagia, including neurovascular accidents (e.g., stroke or head injury), neurodegenerative diseases (e.g., Parkinson’s disease, dementia, amyotrophic lateral sclerosis, multiple sclerosis, or Alzheimer’s disease), neuromuscular problems (e.g., polymyositis/dermatomyositis or myasthenia gravis), and local lesions (e.g., head and neck tumors, surgical resection of the oropharynx/larynx, or radiation injury) [22,23,24]. More than half of all stroke patients and around 30% of traumatic brain injury patients develop some kind of swallowing dysfunction. In addition, approximately 50–80% of patients with Parkinson’s disease, Alzheimer’s disease, and dementia have oropharyngeal dysphagia [12,18,25,26,27]. Many older people also develop oropharyngeal dysphagia [22,23,28,29,30,31]. The prevalence of oropharyngeal dysphagia among institutionalized aged patients is more than 50%, while it is approximately 30% among the general older population [8,9,10,11,12,32,33,34,35].

There is no established pharmacological therapy for the management of oropharyngeal dysphagia [36,37]. Currently, its clinical management is mainly focused on compensatory strategies and swallowing exercises/maneuvers [28,38,39,40]. Common compensatory strategies include modification of the properties of the bolus to be swallowed (e.g., changing the volume, viscosity, or texture of the bolus), and the adoption of different postures before swallowing (e.g., chin tuck or head tilt) [28,38,39,40,41,42,43]. Such compensatory strategies are short-term adjustments that aim to compensate for the swallowing difficulty, but they do not usually change the impaired swallowing physiology or promote the recovery of swallowing function in patients with oropharyngeal dysphagia [38,39,43,44]. Thickeners are often used to increase the viscosity of the bolus, to reduce penetration or aspiration [19,21,45]. Although, increasing the viscosity of the bolus using thickeners can improve swallowing safety, studies have reported that it also increases the amount of oropharyngeal residue [19,21,46,47,48]. Thickeners also have poor palatability, leading to poor compliance by patients [21,46]. Increasing the bolus volume has been reported to increase penetration and aspiration, along with increased amounts of oral [49] and pharyngeal residues, during swallowing in neurogenic oropharyngeal dysphagia patients [19,49]. Some common swallowing exercises/maneuvers include tongue exercises, jaw exercises, effortful swallow exercises, and Mendelsohn maneuvers (voluntarily holding the larynx in an elevated position). The aims of these exercises/maneuvers are to improve the efficacy of swallowing-related muscles, improve the motion of the bolus, and promote modest neuroplastic changes (i.e., the reorganization of neural connections) [39,41,42,43]. Although both compensatory strategies and swallowing exercises/maneuvers are widely used in clinical practice, the evidence to support their effectiveness is often limited [19,21,39,41,42,43,45,50,51,52,53].

In addition to compensatory strategies and swallowing exercises/maneuvers, neurostimulation or sensory stimulation strategies have also been investigated for the management of oropharyngeal dysphagia, although they have not yet become part of mainstream clinical practice [39,41,50,51,52,53,54]. In these strategies, stimuli are applied to central (cortical) or peripheral swallowing-related regions. In central neurostimulation strategies, transcranial magnetic stimulation, or transcranial direct current stimulation is applied to the brain to activate the swallowing-related motor cortex and corticobulbar pathways [39,55,56,57,58,59]. These strategies have shown promising results in stroke patients with oropharyngeal dysphagia [55,56,57,58,60,61]; however, to conduct these therapies (especially transcranial magnetic stimulation), specific and expensive equipment and well-trained professionals are required [62,63]. In peripheral neurostimulation/sensory stimulation strategies, various types of sensory stimuli (e.g., mechanical, thermal, electrical, or chemical) are applied to the oropharyngeal regions. These stimuli increase the sensory inputs to the swallowing center of the brainstem, as well as to the swallowing-related sensory cortex via the sensory nerves that innervate these regions, and thus improve swallowing function [39,54,64,65,66]. Sensory inputs from peripheral swallowing-related regions are important for normal swallowing [66,67,68]. Interrupting sensory inputs from the pharyngeal and laryngeal swallowing-related regions has been reported to disrupt the swallowing process. For example, topical anesthesia applied to the oropharyngeal region disturbs oropharyngeal swallowing and reduces motor cortex activity in the brain [69,70,71]. Moreover, when local anesthesia is applied to the superior laryngeal nerve (SLN), which innervates the laryngopharynx and associated laryngeal regions, healthy individuals have been reported to experience an illusory globus sensation in the throat, effortful swallowing, and laryngeal penetration of fluid during swallowing [72]. Anesthetizing the larynx of healthy adults also results in a higher incidence of penetration, aspiration, and pharyngeal residues when swallowing [73]. In the aged population, sensory input from swallowing-related regions is impaired, and this impairment has been related to oropharyngeal dysphagia and impaired swallowing safety [70,74,75,76,77]. One study reported that, compared with young healthy individuals, the threshold for electrical sensory stimulation to the pharynx is markedly increased in aged healthy individuals, along with a reduction in stimulation-induced activity of the cerebral cortex (pharyngeal event-related potentials) [78]. Additionally, in aged individuals with oropharyngeal dysphagia, there are delayed, impaired, and disruptive patterns of cortical activity in response to pharyngeal stimulation; disturbances in the connections from the throat to the cortex have also been observed [78,79]. Furthermore, studies reported that the older people show delayed onset of the pharyngeal swallow, increased pharyngeal residue, delayed laryngeal vestibule closure, delayed upper esophageal sphincter opening, and delayed hyoid movement during swallowing [6,80,81,82,83]. Sensory deficits in peripheral swallowing-related regions may be caused by reductions in nerve supply in swallowing-related regions in the aged population [74,75,77,84]. Studies have reported that sensory discrimination in the pharyngeal and laryngeal regions progressively diminishes with age [77,84]. Significant sensory deficits in laryngopharyngeal regions have also been observed in stroke and Parkinson’s disease patients [76,85,86].

Because peripheral sensory inputs are important for swallowing, peripheral sensory stimulation strategies have been investigated in animal and clinical studies, and have been found to be effective for modifying swallowing function [39,64,65]. Several review papers have discussed the efficacy of these strategies in the management of oropharyngeal dysphagia [39,54,64,65,66]. Various forms of mechanical stimuli, including touch, pressure, and air puffs, have been investigated and were observed to modulate the swallowing process [54,64,66,87,88]. Thermal sensory stimuli have also been investigated, with diverse results [89,90,91,92,93,94,95,96]. Several studies have used a metal probe to provide cold thermal stimuli, resulting in a combination of cold thermal and mechanical stimuli [90,92,93,96]. Some such studies reported improved swallow responses with this combination of cold thermal and mechanical stimuli [89,91,92,93], while others observed no significant effects [90,95,96]. Electrical stimulation to the pharynx has also been investigated, and was observed to facilitate swallowing function by reducing pharyngeal transit time, swallow response time, and frequency of aspiration [59,97,98,99,100,101,102,103]. Neuromuscular electrical stimulation to activate the peripheral motor nerves supplying swallowing-related muscles has also been investigated; however, inconsistent results were observed [104,105,106,107,108,109]. In addition, transcutaneous electrical sensory stimulation to activate the peripheral sensory nerves (without muscle contraction) leads to a reduced swallow response time and frequency of aspiration in patients with oropharyngeal dysphagia after stroke, but not in patients with Parkinson’s disease [104,110,111,112].

Along with other peripheral sensory stimulation strategies, peripheral chemical sensory stimulation is effective for facilitating swallowing function, and many recent animal and clinical studies have investigated this strategy [44,48,79,82,113,114,115,116,117]. With peripheral chemical sensory stimulation, various chemosensory ion channels that are expressed in peripheral swallowing-related regions are targeted. Chemosensory ion channels can be activated by chemical stimuli and are involved in the transduction of chemical stimuli to neurological signals. In patients with oropharyngeal dysphagia, the activation of these channels by their chemical agonists in peripheral swallowing-related regions results in significant improvements in many of the biomechanical events of swallowing physiology, safety, and efficacy [44,48,49,79,82,113,114,115,118,119]. This strategy is therefore promising for the development of pharmacological therapeutics for oropharyngeal dysphagia [36,37]. The present review discusses recent advancements in peripheral chemical sensory stimulation strategies, including their molecular targets and neurophysiological mechanisms. An understanding of the molecular and neurophysiological mechanisms is important for the development of effective therapeutics.

## 2. Targeting Chemosensory Ion Channels to Improve Swallowing Function

The nerves innervating peripheral swallowing-related regions are reported to respond to chemical stimuli [120,121,122,123]. A number of studies, including by our group, have observed that the afferent nerves innervating the pharyngeal and laryngopharyngeal regions respond to various acids (e.g., citric acid and HCl) and salts (e.g., KCl and NH_4_Cl) [120,121,122,123]. The second-order neurons, located in the nucleus tractus solitarii (NTS), also respond to chemical stimuli applied to peripheral swallowing-related regions [124]. These observations suggest that various chemosensory ion channels in peripheral swallowing-related regions may be activated by chemical stimuli. Moreover, promising results have been observed when these channels are targeted with their chemical agonists to improve swallowing function (Table 1 and Table 2).

### 2.1. Targeting Transient Receptor Potential Channels (TRPs)

TRPs are integral membrane proteins of the plasma membrane that act primarily as non-selective ion channels [125,126]. Many TRPs are polymodal and can be activated by various stimuli, including thermal, mechanical, and chemical stimuli [125,126,127]. The polymodal nature of these channels make them ideal molecular interfaces between a range of external stimuli and the nervous system. Their expression has been observed in both neuronal and non-neuronal tissues [125,126,127], and they play an important role in many physiological and pathological processes [125,126,127,128,129]. To date, 28 mammalian TRPs have been cloned, and they can be grouped on the basis of their amino acid sequence homology into six subfamilies: TRPA (ankyrin), TRPC (canonical), TRPM (melastatin), TRPML (mucolipin), TRPP (polycystin), and TRPV (vanilloid) [125,126,129]. Chemical agonists of several members of these TRPs have been used in swallowing-related research to understand their effects on swallowing physiology, as well as their therapeutic effects in the management of oropharyngeal dysphagia (Table 1 and Table 2).

**Table 1:** Animal studies investigating the effects of targeting chemosensory ion channels on swallowing.

Targeting Channels	Agonists and Its Application	Animals	Mode of Application	Effects on Swallowing	Ref.
**TRPV1**	Capsaicin solution (25 μM) into the laryngopharynx and associated laryngeal regions	Rats	Acute	Capsaicin triggered a greater number of swallowing reflexes compared to distilled water/saline/vehicle; Capsaicin shortened the intervals between the evoked swallowing reflexes compared to distilled water/saline/vehicle; Prior topical application of a TRPV1 antagonist significantly reduced the number of capsaicin-induced swallowing reflexes and lengthened the intervals between the evoked reflexes.	[116]
	Capsaicin solution (10 μM) into the larynx	Guinea pigs	Acute	Capsaicin triggered a greater number of swallowing reflexes compared to saline.	[130]
	Capsaicin solution (10 μM) on the vocal folds	Rats	Acute	Capsaicin triggered a considerable number of swallowing reflexes.	[131], [132]
	Capsaicin solution (600 nM) into the pharyngolaryngeal region	Rats (a dysphagia model)	Acute	Capsaicin improved the triggering of swallowing reflexes compared to that of distilled water.	[133]
**TRPM8**	Menthol solution (50 mM) into the laryngopharynx and associated laryngeal regions	Rats	Acute	Menthol triggered a greater number of swallowing reflexes compared to distilled water/saline/vehicle; Menthol shortened the intervals between the evoked reflexes compared to distilled water/saline/vehicle; Prior topical application of a TRPM8 antagonist significantly reduced the number of menthol-induced swallowing reflexes and lengthened the intervals between the evoked reflexes.	[116]
**ASIC3**	Guanidine-4-methylquinazoline (GMQ) solution (0.5 to 10 mM) into the laryngopharynx and associated laryngeal regions	Rats	Acute	GMQ dose-dependently facilitated the triggering of swallowing reflex; Prior topical application of an ASIC3 antagonist significantly reduced the number of GMQ-induced swallowing reflexes and lengthened the intervals between the evoked reflexes.	[117]
	Agmatine (50 mM to 2 M) solutions into the laryngopharynx and associated laryngeal regions	Rats	Acute	Agmatine dose-dependently facilitated the triggering of swallowing reflex; Prior topical application of an ASIC3 antagonist significantly reduced the number of agmatine-induced swallowing reflexes and lengthened the intervals between the evoked reflexes.	[117]
**ASICs and TRPV1**	Acetic acid (5 to 30 mM), citric acid (5 to 30 mM) solutions into the pharyngolaryngeal region	Rats	Acute	Acetic acid and citric acid evoked a greater number of swallowing reflexes compared to distilled water.	[134]
	Citric acid solution (10 mM) into the pharyngolaryngeal region	Rats (a dysphagia model)	Acute	Citric acid solution improved the triggering swallowing reflexes compared to that of distilled water.	[133]

Note: Acute application refers to the condition when the agonists applied for a single time in the swallowing-related regions.

**Table 2:** Human studies investigating the effects of targeting chemosensory ion channels on swallowing.

Targeting Channels	Agonists and Its Application	Patients/Participants	Mode of Application	Effects on Swallowing	Ref.
**TRPV1**	Capsaicin (1 nM to 1 μM) solution into the pharyngeal region	Aged patients with cerebrovascular diseases or dementia presenting oropharyngeal dysphagia	Acute	Capsaicin solution dose-dependently reduced the latency to trigger a swallow response.	[118]
	Capsaicinoid (150 μM) containing nectar bolus ingestion	Aged patients presenting oropharyngeal dysphagia	Acute	Laryngeal vestibule closure time during swallowing reduced; Upper esophageal sphincter opening time during swallowing reduced; Time for maximal vertical movement of the hyoid bone and larynx during swallowing reduced; Prevalence of laryngeal penetration during swallowing reduced; Prevalence of pharyngeal residue of bolus during swallowing reduced.	[44]
	Capsaicinoid (150 μM) containing nectar bolus ingestion	Aged/stroke/neurodegenerative disease patients presenting oropharyngeal dysphagia	Acute	Laryngeal vestibule closure time during swallowing reduced; Prevalence of laryngeal penetration during swallowing reduced; Prevalence of pharyngeal residue of bolus during swallowing reduced; Bolus propulsion velocity during swallowing increased.	[48]
	Capsiate (1–100 nM) into the pharyngeal region	Patients with history of aspiration pneumonia presenting oropharyngeal dysphagia	Acute	Capsiate dose-dependently reduced the latency to trigger a swallow response.	[135]
	Capsaicinoid (10 μM) containing nectar bolus ingestion	Aged patients presenting oropharyngeal dysphagia	Chronic (three times/day, before meals for 10 days)	Laryngeal vestibule closure time during swallowing reduced; Score of the penetration-aspiration scale lowered; Amplitude of cortical sensorial response to pharyngeal electrical stimulation increased; Latency to evoke cortical sensorial response to pharyngeal electrical stimulation decreased.	[79]
	Capsaicin containing pickled cabbage (1.5 μg/10 g) ingestion	Healthy participants	Chronic (before every major meal/day for 20 days)	Latency to trigger a swallow response reduced	[136]
	Capsaicin containing lozenges (1.5 μg/lozenge)	Aged patients with cerebrovascular diseases presenting oropharyngeal dysphagia	Chronic (before every major meal/day for 4 weeks)	Latency to trigger a swallow response reduced.	[119]
	Capsaicin containing thin film food (0.75 μg/film) ingestion	Aged patients presenting oropharyngeal dysphagia	Chronic (before every major meal/day for 1 week)	Duration of cervical esophageal opening during swallowing shortened; Symptoms of oropharyngeal dysphagia reduced; Substance P concentration in saliva increased in patients who showed improvement of swallowing.	[113]
	Capsaicin (150 μM) containing nectar bolus ingestion along with cold thermal tactile stimulation	Aged patients with history of stroke presenting oropharyngeal dysphagia	Chronic (three times/day, before meals for 3 weeks)	Swallowing function improved assessed by swallowing assessment tools.	[137]
	Capsaicinoid (10 μM) containing nectar bolus ingestion	Aged patients presenting oropharyngeal dysphagia	Chronic (three times/day, before meals for 10 days)	The swallowing safety improved evidenced by reduction of the prevalence of aspiration and lowering the score in penetration-aspiration scale.	[114]
	Capsaicin (0.5 g of 0.025%) containing ointment into the ear canal	Aged patients presenting oropharyngeal dysphagia	Acute and chronic (once daily for 7 days)	Swallowing function improved.	[138]
**TRPM8**	Menthol solution (100 μm to 10 mM) into the pharyngeal region	Aged patients presenting oropharyngeal dysphagia	Acute	Menthol dose-dependently reduced the latency to trigger a swallow response.	[139]
	Menthol (1 and 10 mM) containing nectar bolus ingestion	Aged/stroke/neurodegenerative diseases patients presenting oropharyngeal dysphagia	Acute	Laryngeal vestibule closure time during swallowing reduced; Prevalence of laryngeal penetration during swallowing reduced.	[48]
**TRPA1**	Cinnamaldehyde (756.6 μM) and zinc (70 μM) containing nectar bolus ingestion	Aged/stroke/neurodegenerative diseases patients presenting oropharyngeal dysphagia	Acute	Laryngeal vestibule closure time during swallowing reduced; Upper esophageal opening time during swallowing reduced; Score in penetration-aspiration scale lowered; Frequency of safe swallows increased; Latency of evoking cortical response to pharyngeal electrical stimulation shortened.	[82]
	Citral (1.6 mM) containing nectar bolus ingestion	Aged/stroke/neurodegenerative diseases patients presenting oropharyngeal dysphagia	Acute	Laryngeal vestibule closure time during swallowing reduced; Upper esophageal opening time during swallowing reduced.	[82]
**TRPV1 and TRPA1**	Piperine (150 μM and 1 mM) containing nectar bolus ingestion	Aged/stroke/neurodegenerative diseases patients presenting oropharyngeal dysphagia	Acute	Laryngeal vestibule closure time during swallowing reduced; Time required for maximum anterior extension of hyoid bone during swallowing reduced; Score in penetration aspiration scale lowered; Prevalence of laryngeal penetration during swallowing reduced.	[115]
	Black pepper oil (a volatile compound) (100 μL for 1 min) to the nostrils with a paper stick for inhalation.	Aged patients with cerebrovascular diseases presenting oropharyngeal dysphagia	Acute	Latency to trigger a swallow response for distilled water reduced.	[140]
	Piperine (150 μM and 1 mM) containing nectar bolus ingestion	Aged/stroke/neurodegenerative diseases patients presenting oropharyngeal dysphagia	Acute	Laryngeal vestibule closure time during swallowing reduced; Prevalence of penetration during swallowing reduced; Bolus propulsion velocity during swallowing increased.	[48]
	Black pepper oil (a volatile compound) (100 μL for 1 min) to the nostrils with a paper stick for inhalation.	Aged patients with cerebrovascular diseases presenting oropharyngeal dysphagia	Chronic (three times/day, before meals for 30 days)	Latency to trigger a swallow response for distilled water reduced; Serum substance P level increased; Regional cerebral blood flow in right orbitofrontal and left insular cortex increased.	[140]
	Black pepper oil (a volatile compound) (100 μL for 1 min) to the nostrils with a paper stick for inhalation.	Pediatric patients with severe neurological disorders often receiving tube feeding	Chronic (three times/day, before meals for 3 months)	The amount of oral intake of foods by the patients increased; Swallowing-related movements increased.	[141]
**TRPV1, TRPA1 and TRPV3**	Vanillin (a volatile compound), (flow rate 7 L/min for 200 ms) delivered ortho-and retro-nasally	Healthy participants	Acute	The frequency of swallowing for continuous intraoral sweet stimuli (glucose) increased in case of retro-nasal delivery.	[142]
**TRPA1 and TRPM8**	Citral (1.6 mM) and isopulegol (1.3 mM) containing nectar bolus ingestion	Aged/stroke/neurodegenerative diseases patients presenting oropharyngeal dysphagia	Acute	Upper esophageal opening time during swallowing reduced.	[82]
**ASICs and TRPV1**	Citric acid (2.7% or 128 mM) containing liquid bolus ingestion	Aged patients with neurological diseases presenting oropharyngeal dysphagia	Acute	Prevalence of aspiration and penetration during swallowing reduced.	[143]
	Lemon juice containing barium liquid bolus (1:1) ingestion	Patients with strokes and neurological diseases presenting oropharyngeal dysphagia	Acute	Swallow onset time reduced; Time required to trigger the pharyngeal swallow (pharyngeal delay time) reduced; Frequency of aspiration reduced; Oropharyngeal swallow efficiency increased.	[49]
	Lemon juice containing barium liquid bolus (1:1) ingestion	Healthy participants and head and neck cancer patients	Acute	Pharyngeal transit time reduced.	[144]
	Citric acid (80 mM) delivered on the tongue	Healthy participants	Acute	Frequency of swallowing increased; Hemodynamic responses in the cortical swallowing-related areas prolonged.	[145]
	Lemon juice application on the tongue along with nasal inhalation of lemon juice odor	Healthy participants	Acute	Motor evoked potential from the submental muscles increased during volitional swallowing induced by transcranial magnetic stimulation.	[146]
	Citric acid solution (20 mM) ingestion	Healthy participants	Acute	Activity of submental muscle during swallowing increased.	[147]
	Citric acid solution (2.7% or 128 mM) ingestion	Healthy participants	Acute	Amplitude of anterior tongue-palate pressure during swallowing increased; Activity of submental muscles during swallowing increased.	[148]
	Lemon juice (10%) solution ingestion (4 °C before delivery)	Healthy participants and stroke patients with and without oropharyngeal dysphagia	Acute	Inter-swallow interval shortened in healthy participants of <60 years of age; Inter-swallow interval unaffected in stroke patients; Velocity and capacity of swallowing reduced both in healthy individuals and stroke patients.	[149]
	Lemon juice delivered on tongue	Healthy participants	Acute	Number of swallowing increased; Salivation increased; Amount of salivation correlated with the number of swallowing.	[150]
	Acetic acid (10 and 100 mM) applied on the posterior part of the tongue	Healthy participants	Acute	Latency to trigger swallowing prolonged compared to that of water.	[151]
	Citric acid (2.7%) solution ingestion	Healthy participants	Acute	Lingual pressure during swallowing increased.	[152]
	Citric acid (10%) solution ingestion	Healthy participants	Acute	Speed of swallowing reduced compared to that of water.	[153]
	Citric acid containing gelatin cubes (4.4 g of citric acid in 200 ml of gelatin) chewing and ingestion	Healthy participants	Acute	Oral preparation time during swallowing accelerated; Amplitude of submental muscle activity during swallowing increased; Duration of submental muscle activity during swallowing reduced.	[154]
	Lemon water (50%) solution ingestion	Healthy participants	Acute	Activity of submental muscles during swallowing increased; Onset time of activation of the submental muscles closely approximated.	[155]
	Lemon juice (a drop of 100% lemon juice in the anterior faucial pillar) + cold mechanical stimuli using a probe (around 8–9 °C) before swallowing of water	Healthy participants	Acute	Latency to trigger swallowing reduced.	[156]
	Lemon juice (1:16, mixed with water) ingestion	Healthy participants	Acute	Onset time of activation of the submental and infrahyoid muscles shortened.	[157]

Note: Chronic application refers to the condition when the agonists applied for multiple times over a period of time in the swallowing-related regions.

#### 2.1.1. Targeting TRPV1

TRPV1 was the first member of the TRPV subfamily to be isolated [158]. It can be activated by a wide range of natural compounds (e.g., capsaicin, capsiate, piperine, resiniferatoxin, tinyatoxin, camphor, eugenol, gingerols, shogaols, cannabidiol, carvacrol, evodiamine, vanillin, and thymol), synthetic compounds (e.g., olvanil and arvanil), acids/low pH, and thermal stimuli (~43 °C) [125,126,158,159,160,161,162,163,164,165,166]. It is also activated by endogenous ligands such as protons, anandamide, arachidonic acid metabolites, and N-arachidonyl dopamine [125,126,127,161].

Studies have reported TRPV1 expression in swallowing-related regions. In animal studies, TRPV1 immunoreactivity has been observed in the intraepithelial and subepithelial nerve fibers of the oral cavity, tongue, soft palate, pharynx, epiglottis, trachea, and larynx [167,168,169,170]. Immunoreactivity has also been observed in the oral and olfactory epithelium [169,171,172,173,174], and within and beneath the taste papillae located in the tongue, soft palate, and epiglottis [167,169]. In addition, TRPV1 is also expressed in peripheral ganglia (e.g., the trigeminal, nodose, petrosal, and jugular ganglia) [116,128,167,175,176,177], which contain the cell bodies of afferent neurons that carry sensory inputs from peripheral swallowing-related regions. In the nodose and petrosal ganglia of rats, around one-third of retrogradely traced afferent neurons from the pharyngeal and soft palate regions show TRPV1 immunoreactivity [167]. We have also observed that around one-third to one-half of the retrogradely traced afferent neurons from the laryngopharyngeal and associated laryngeal regions showed TRPV1 immunoreactivity in the nodose, petrosal, and jugular ganglionic complex (NPJc) [116]. Approximately two-thirds of these neurons were unmyelinated [116]. In humans, TRPV1 expression has been observed in epithelial cells and subepithelial nerve fibers of the tongue, pharynx, nasal cavity, epiglottis, and larynx [178,179,180]. The presence of TRPV1 channels in swallowing-related regions provides evidence of their involvement in the swallowing process.

##### Effects of TRPV1 Agonists on Swallowing

The effects of chemical TRPV1 agonists on swallowing processes have been investigated in animals (Table 1) and humans (Table 2). In animals, the topical application of capsaicin (a natural pungent ingredient of chili) to swallowing-related regions facilitates the triggering of the swallowing reflex [116,130,131,132]. We have previously reported that using a capsaicin-containing solution to stimulate the laryngopharynx and associated laryngeal regions in rats leads to increased numbers of evoked swallowing reflexes compared with vehicle, saline, or distilled water [116]. Capsaicin application also shortens the intervals between evoked reflexes [116]. Furthermore, the topical application of a TRPV1 antagonist prior to the application of capsaicin significantly reduces the number of swallowing reflexes and lengthens the intervals between the evoked reflexes, indicating the specific involvement of TRPV1 [116]. Additionally, the use of different concentrations of capsaicin modulated the SLN response that innervates the laryngopharynx and associated laryngeal regions [116]. In an animal model of dysphagia induced by transient middle cerebral artery occlusion, the TRPV1 agonist capsaicin has also been observed to overcome the reduced ability to trigger swallowing reflexes [133].

In humans, a number of studies have investigated the efficacy of TRPV1 agonists to modulate swallowing behavior. The acute or chronic ingestion of TRPV1 agonist-containing solutions, foods, or boluses modulates the various biomechanical events of swallowing (Table 2). In patients with dysphagia associated with cerebral thrombosis or dementia, the acute application of a capsaicin-containing solution (1 nM to 1 µM) to the pharyngeal region causes dose-dependent reductions in the latency to trigger the swallow response [118]. Another study found that the acute ingestion of nectar boluses containing 150 µM capsaicinoid (present in hot chili sauce), prepared using a thickener, markedly improved swallowing safety in older dysphagic patients with oropharyngeal dysphagia. This improvement occurred via shortening the time for laryngeal vestibule closure and upper esophageal sphincter opening, and by shortening the time to the maximal vertical movement of the hyoid and larynx, compared with the ingestion of nectar boluses without capsaicinoid. Capsaicinoid ingestion also markedly reduced the prevalence of laryngeal penetration and pharyngeal residue in this study [44]. However, the acute ingestion of a lower dose (10 µM) of capsaicinoid-containing nectar boluses did not exert significant changes in swallowing events in chronic poststroke and older patients with oropharyngeal dysphagia [79,181], although it did increase the excitability of the motor cortex in response to pharyngeal electrical stimulation [181]. Another study reported that the acute application of red wine (without alcohol) or the polyphenols obtained from Cabernet Sauvignon grapes (used to make red wine) to the pharyngeal region led to reduced latencies of swallow responses in older patients with dysphagia associated with cerebrovascular diseases [182]. This effect may be caused by the positive allosteric actions of the polyphenols on TRPV1 [182]. The same study found that polyphenols increased capsaicin-induced currents in small-diameter dorsal root ganglion neurons inhibited by a TRPV1 antagonist (capsazepine) in mice. However, the polyphenols themselves did not increase currents in the neurons, suggesting the positive allosteric action of polyphenols on capsaicin-induced TRPV1 activation [182].

The effects of chronic TRPV1 agonist ingestion on swallowing have been investigated both in healthy participants without oropharyngeal dysphagia and in patients with oropharyngeal dysphagia with different etiologies (Table 2). In healthy older and young participants with no swallowing difficulties, the chronic supplementation of capsaicin-containing pickled cabbage before every major meal for 20 days reduced the latency to evoke swallowing in response to a glucose solution delivered to the pharynx [136]. One week after the supplementation had ended, the effects of capsaicin supplementation remained in young participants but had faded out in older participants [136]. In a mid-term randomized controlled study, older patients with dysphagia associated with cerebrovascular diseases received chronic daily supplementation of lozenges containing a low concentration of capsaicin (1.5 µg/lozenge) before every major meal for 4 weeks [119]. Compared with a placebo, this supplementation significantly reduced the latency to evoke swallowing in response to distilled water delivered to the pharynx [119]. The extent of the reduction in latency was greater in patients who had a long baseline latency to evoke swallowing before starting the supplementation [119]. Participants in this study did not complain of unpleasant feelings or show symptoms of any clinical complications related to the supplementation, either during the study period or for several months after the investigation [119]. In a double-blind, placebo-controlled, crossover study conducted in older patients with oropharyngeal dysphagia, patients received chronic daily supplementation of capsaicin in film foods before every major meal for 1 week. Compared with a placebo, this treatment improved the symptoms of oropharyngeal dysphagia and shortened the duration of cervical esophageal opening in a greater number of patients [113]. In addition, in the patients who had improved swallow responses to the capsaicin supplementation, the neuropeptide substance P was significantly increased in saliva after capsaicin administration compared with placebo, even though the amount of saliva was unchanged [113]. A randomized, double-blind study was conducted in older stroke patients with oropharyngeal dysphagia [137]. Supplementation with capsaicin-containing nectar boluses along with cold thermal and tactile stimuli before every major meal for 3 weeks improved swallowing function, as assessed using swallowing assessment tools (the water swallow test and eating assessment tool), compared with placebo [137]. No adverse reactions were attributed to capsaicin supplementation in this study [137]. Another randomized study was conducted to compare the effects of mid-term (5 days/week for 2 weeks) capsaicinoid supplementation (three times/day, before meals) and transcutaneous sensory electrical stimulation (1 h/day) in older patients with oropharyngeal dysphagia [114]. Both treatment strategies improved the safety of swallowing by reducing aspiration, with lower scores in the penetration–aspiration scale [114]. However, capsaicin treatment was effective in a greater percentage of patients (approximately 68%) compared with transcutaneous sensory electrical stimulation therapy (approximately 42%). The authors reported no serious adverse events related to either capsaicin or transcutaneous sensory electrical stimulation therapy [114]. Another study compared the effects of the acute and chronic ingestion of capsaicinoid-containing nectar boluses on swallowing function, as well as on cortical activity (sensorial event-related potential) in response to pharyngeal electrical stimulation (using electroencephalography) [79]. This study observed that the acute application of 10 μM capsaicinoid-containing nectar boluses to aged patients did not improve swallowing function or exert significant changes in pharyngeal electrical stimulation-induced cortical activity [79]. However, chronic supplementation with the same amount of capsaicinoid reduced the impaired safety of swallowing compared with placebo supplementation [79]. Additionally, chronic supplementation with the TRPV1 agonist improved cortical sensorial responses to pharyngeal electrical stimulation compared with placebo supplementation [79]. These results indicate improvements in the conduction and integration of sensory information in the cortex [79]. Furthermore, in the patients receiving chronic supplementation, the reductions in laryngeal vestibule closure time were strongly correlated with reductions in the latency to evoke a cortical response to pharyngeal electrical stimulation [79]. This finding indicates a relationship between improved cortical activity and improved swallowing function [79]. The findings of this study suggest that chronic TRPV1 stimulation in peripheral swallowing-related regions leads to neuroplastic changes in the cerebral cortex that augment any improvements in swallowing function [79]. The authors of this study also reported that chronic TRPV1 stimulation did not produce any adverse events or desensitization effects in patients [79].

To avoid pungency of capsaicin/capsaicinoids, a non-pungent agonist of TRPV1, capsiate, was used in dysphagic patients with a history of aspiration pneumonia to assess its effects on swallowing function [135]. Acute application of capsiate (1–100 nM) to the pharyngeal region through a nasal catheter caused dose-dependent reductions in the latency to evoke a swallowing [135]. At doses of 10 and 100 nM, the latencies were significantly shorter than that of distilled water [135].

Application of a TRPV1 agonist into the ear canal can also improve swallowing performance [138]. A pilot study reported that acute or chronic (once daily for 7 days) application of capsaicin-containing ointment (around 1 mM) in the external auditory canal improved swallowing function in older patients with dysphagia, as assessed by endoscopic swallowing scores [138]. In this study, the effects lasted for 60 min after an acute application [138]. Moreover, chronic application led to significantly improved swallowing function in patients with severe swallowing problems [138]. The effects observed in this study may be attributed to capsaicin-induced activation of the auricular branch of the vagus nerve (Arnold’s nerve), and the subsequent ectopic antidromic release of substance P in laryngopharyngeal regions [138].

TRPV1 agonists have also been reported to modulate upper gastrointestinal tract motility [183,184,185]. In healthy individuals, the acute application of capsaicin to the esophagus improves esophageal clearance by increasing the strength of primary (swallow-induced) [183] and secondary esophageal peristalsis [186], and by lowering esophageal sphincter pressure [183].

#### 2.1.2. Targeting TRPA1

TRPA1 was identified slightly later than TRPV1 [187,188]. TRPA1 can be activated by a wide range of natural and synthetic chemical stimuli, such as allyl isothiocyanate (present in mustard oil and wasabi) [189,190], cinnamaldehyde (present in cinnamon oil) [190], allicin and diallyl disulfide (present in garlic extract) [191,192,193], methyl salicylate (present in wintergreen oil) [190], gingerol (present in ginger) [194], carvacrol (present in oregano) [195], curcumin (present in turmeric) [196], umbellulone (present in *Umbellularia californica*) [197], ligustilide (present in *Angelica acutiloba*) [198], heavy metals (e.g., zinc, copper, or cadmium) [199,200], tetrahydrocannabinol [189], formalin [201], lipid peroxidation products (e.g., prostaglandin) [202], and oxidative stress products (e.g., hydrogen peroxide or 4-hydroxynonenal) [203,204,205]. Volatile compounds and odorants (e.g., ethyl vanillin, α-terpineol, or amyl acetate) can also activate TRPA1 channels [206,207,208]. 

Expression of TRPA1 has been observed on nerve fibers and in epithelial cells in the oral, nasal, pharyngeal, laryngeal, and esophageal regions [173,209,210,211]. TRPA1 is also expressed in the trigeminal, nodose, jugular, and petrosal ganglia [128,212,213,214]. In human biopsy tissues from oropharyngeal regions, TRPA1 was observed to be localized on submucosal structures, including nerve fibers and cells resembling fibroblasts [178].

##### Effects of TRPA1 Agonists on Swallowing

The effects of the acute administration of TRPA1 agonists on safety, efficacy, and the biomechanical events of swallowing were investigated in a three-arm, quadruple-blind, randomized clinical trial that included patients with oropharyngeal dysphagia associated with aging, stroke, or neurodegenerative diseases [82]. The patients received nectar boluses mixed with citral (1.6 mM) and a combination of cinnamaldehyde (756.6 μM) and zinc (70 μM) as TRPA1 agonists, and a combination of citral (1.6 mM) and isopulegol (1.3 mM) as a mix of TRPA1 and TRPM8 agonists [82]. All of these agonists significantly reduced the upper esophageal sphincter opening time. The TRPA1 agonists (cinnamaldehyde and zinc combination, and citral) significantly reduced the laryngeal vestibule closure time, but the combination of TRPA1 and TRPM8 agonists (citral and isopulegol combination) did not [82]. The cinnamaldehyde and zinc combination also reduced the penetration-aspiration scale scores and increased the frequency of safe swallows in patients with oropharyngeal dysphagia [82]. Moreover, the cinnamaldehyde and zinc combination reduced the latency to evoke a cortical response to pharyngeal electrical stimulation [82]. The cinnamaldehyde and zinc combination was also observed to be the most efficient, safe, palatable, and well-tolerated among the agonists [82]. Citral was reported to be more intense and less pleasant than the placebo when the patients were asked to rate the palatability of the agonists. No adverse events or severe adverse events were reported to be related to agonist use in this study [82].

#### 2.1.3. Effects of Dual TRPV1 and TRPA1 Agonists on Swallowing

The stimulation of swallowing-related regions with a dual TRPV1 and TRPA1 agonist, piperine, has been observed to improve swallowing function in patients with oropharyngeal dysphagia [48,115]. A randomized, double-blind, controlled study was conducted in older patients with oropharyngeal dysphagia related to aging, neurodegenerative diseases, or stroke. In this study, the acute ingestion of piperine-containing nectar boluses (150 μM and 1 mM) significantly improved swallowing safety compared with piperine-free nectar bolus ingestion [115]. However, the prevalence of oropharyngeal residues, maximal vertical and anterior distances of hyoid movement, and speed of bolus propulsion were not significantly affected by piperine treatment in this study. Abdominal pain occurred in one participant, but it was found to not be related to piperine administration [115]. The results of another study, involving the same kinds of patients with oropharyngeal dysphagia, also supported the efficacy of piperine for improving swallowing safety [48].

The nasal inhalation of piperine also improves swallowing behavior. A randomized, controlled study involving aged patients with a previous history of stroke investigated the acute nasal inhalation of black pepper oil (100 μL for 1 min, administered to the nostrils with a paper stick) [140]. Compared with both lavender oil and distilled water, this treatment significantly reduced the latency to evoke a swallowing in response to distilled water delivered to the pharyngeal region [140]. In addition, compared with pretreatment latencies, the chronic inhalation of black pepper oil (three times/day, before each meal), but not lavender oil, for 30 days significantly reduced the latency to evoke swallowing [140]. Serum substance P levels and regional cerebral blood flow in the right orbitofrontal and left insular cortices were also increased in patients who received chronic inhalation of black pepper oil [140]. Another study investigated the effects on swallowing behavior in healthy individuals of orthonasal (in the external nares) and retronasal (in the nasopharynx) delivery of a food flavor compound, vanillin (flow rate: 7 L/min for 200 ms), combined with continuous intraoral sweet stimuli (glucose) [142]. There was an increased frequency of swallowing with reduced latency when vanillin was delivered retronasally compared with orthonasal delivery [142]. This finding suggests that retronasally presented odorants may influence swallowing function [142]. It has recently been reported that vanillin can activate TRPV1, TRPA1, and TRPV3 channels [159,161,195]. Thus, the vanillin-induced activation of these channels in the nasopharyngeal mucosa or olfactory epithelium may cause the observed effects on swallowing. Furthermore, another study investigated the effects of chronic nasal inhalation (100 μL for 1 min before meals, for 3 months) of black pepper oil in eight pediatric patients with severe neurological disorders who often received tube feeding [141]. Five of the eight patients responded positively to the chronic inhalation of black pepper oil, with increased amounts of oral food intake and swallowing-related movements [141].

#### 2.1.4. Targeting TRPM8

TRPM8 can be activated by various natural chemical agents, such as menthol, eucalyptol, linalool, and isopulegol, or by synthetic chemical ligands such as icilin [215]. It is also activated by mildly cool to noxiously cold temperatures [216,217,218]. TRPM8 expression has been observed in swallowing-related regions and ganglia [116,212,219,220,221,222,223,224,225,226]. Animal studies have reported TRPM8 expression on nerve fibers, epithelial cells, and taste buds in the oral mucosa, nasal mucosa, soft palate, pharynx, larynx, and epiglottis [219,221,222,223,224]. In human biopsy tissue from oropharyngeal regions, TRPM8 expression has been observed in the afferent nerve fibers that innervate the mucosa of the human tongue, pharynx, and lingual surface of the epiglottis [227]. The cell bodies of afferent neurons from these regions also express TRPM8 [116,212,219,222]. In an animal study by our group, we observed that two-thirds of the TRPM8-immunoreactive SLN afferent neurons in the NPJc were unmyelinated in rats [116].

##### Effects of TRPM8 Agonists on Swallowing

The application of TRPM8 agonists to swallowing-related regions facilitated the evoking of the swallowing reflex in our animal study [116]. The acute application of a TRPM8 agonist, menthol, to the laryngopharynx and associated laryngeal areas evoked a significantly greater number of swallowing reflexes with shortened inter-swallow intervals compared with vehicle, saline, or distilled water. These results suggest a facilitated triggering of the swallowing reflex when TRPM8 is activated [116]. Moreover, the topical application of a TRPM8 antagonist prior to applying the menthol solution led to a significantly reduced number of swallowing reflexes and lengthened intervals between the evoked reflexes, indicating the specific involvement of TRPM8 in the observed effect. Additionally, different concentrations of menthol modulated the sensory nerve responses that carry information from the stimulated regions [116].

A clinical study conducted in institutionalized older patients with mild to moderate swallowing difficulties revealed that the acute application of menthol solution (100 μm to 10 mM) to the pharyngeal region led to dose-dependent reductions in the latency to evoke a swallowing [139]. No adverse effects or unpleasant feelings were reported by patients during or after the application of menthol in this study [139].

#### 2.1.5. Comparison of the Effects of Different TRP Agonists on Swallowing

The therapeutic effects of the acute ingestion of nectar boluses containing a TRPV1 agonist (capsaicinoids, 150 μM), a dual TRPV1 and TRPA1 agonist (piperine, 150 μM and 1 mM), and a TRPM8 agonist (menthol, 1 and 10 mM) were compared in patients with oropharyngeal dysphagia associated with aging, stroke, or neurodegenerative diseases [48]. All of these agonists improved swallowing safety by reducing the prevalence of penetration and the laryngeal vestibule closure time [48]. Only the TRPV1 agonist improved the swallowing efficacy by reducing the prevalence of pharyngeal residue and increasing bolus propulsion speed [48]. The TRPV1 agonist-containing bolus showed the greatest therapeutic effects for improving swallowing efficacy and safety, while the TRPM8 agonist showed the weakest therapeutic effects [48]. In our animal study, we also observed that a TRPV1 agonist (capsaicin) facilitated the triggering of the swallowing reflex at lower concentrations than a TRPM8 agonist (menthol), suggesting the greater efficacy of the TRPV1 agonist [116].

#### 2.1.6. Stepwise Therapy Using Different TRP Agonists

A study of stepwise therapy using TRP agonists was conducted in dysphagic patients with a history of recurrent pneumonia. In this study, patients received black pepper oil aromatherapy followed by lozenges containing capsaicin (three times daily for 5 days) and jelly with menthol (one time daily), in a stepwise manner. When the patients were able to safely swallow the menthol jelly, they were provided with food with different textures (e.g., paste or pudding textures, or regular meals). The stepwise method was effective for decreasing the incidence of pneumonia, presumably by improving swallowing, leading to reduced aspiration [228].

### 2.2. Targeting Acid-Sensing Ion Channels (ASICs)

ASICs are members of the degenerin/epithelial sodium channel (DEG/ENaC) family, and allow the entry of cations (mainly Na^+^) into ASIC-expressing cells upon activation [229,230,231]. ASICs are generally activated by acids. They have several subunits: ASIC1a, ASIC1b ASIC2a, ASIC2b, ASIC3, and ASIC4 [229,230,231]. Most of these subunits are expressed in both the central and the peripheral nervous system, although ASIC1b and ASIC3 are predominantly detected in the peripheral nervous system [229,230,231]. The expression of various ASICs has been observed on neurons present in the trigeminal, vagal, and glossopharyngeal ganglia [232,233,234,235]. They are expressed on taste buds and epithelial cells of the tongue [236,237], as well as on nerve fibers and epithelial cells in the esophagus [233,238,239]. In our animal study, we observed ASIC3 on epithelial cells and afferent nerve fibers in the laryngopharynx and associated laryngeal regions innervated by the SLN [117]. Moreover, in human biopsy tissues of oropharyngeal regions, ASIC3 expression has been observed in the afferent nerve fibers that innervate the mucosa of the human tongue, pharynx, and lingual surface of the epiglottis [227]. ASICs are also expressed in human nasal epithelium [240].

#### Effects of ASIC Agonists on Swallowing

Weak acids (e.g., citric acid) and sour-tasting substances containing weak acids (e.g., lemon juice) have been studied to investigate their effects on swallowing (Table 1 and Table 2). In an animal study, we observed that the topical acute application of citric acid or acetic acid in the pharyngolaryngeal regions facilitated the triggering of swallowing reflexes compared with distilled water [134]. Acids can activate both TRPV1 and ASICs. To understand the specific involvement of ASIC channels in swallowing, we recently used non-acid/non-proton activators for ASIC3 in an animal study [117]. The topical application of a natural (agmatine) and a synthetic (guanidine-4-methylquinazoline) non-proton ASIC3 agonist into the laryngopharynx and associated laryngeal regions dose-dependently facilitated the triggering of swallowing reflexes [117]. This faciliatory effect of ASIC3 was significantly suppressed by the prior topical application of an ASIC3 antagonist, suggesting the specific involvement of these channels in the facilitation [117].

In human studies, sour-tasting substances or weak acids have been incorporated into solutions or boluses and presented to the oral cavity or pharyngolaryngeal regions (Table 2). This experimental methodology allows the activation of sour taste receptors, ASICs, and other acid-activating channels in these regions.

Lemon juice, which contains citric acid, has been used in several studies to investigate its effects on swallowing and on the activity of swallowing-related muscles in healthy individuals and patients with oropharyngeal dysphagia [150,155,157]. The application of lemon juice on the tongue increases salivation and the frequency of swallowing in healthy adults, and swallowing frequency is correlated with the amount of salivation after lemon juice application [150]. In addition, increased electromyographic activity [155] and an earlier onset of action in the submental and infrahyoid muscles has been observed in healthy individuals during the ingestion of water mixed with lemon juice [155,157]. Another study mixed lemon juice with barium liquid boluses (1:1) to assess their effects on swallowing in patients with oropharyngeal dysphagia associated with stroke and neurological diseases [49]. In this study, the bolus mixed with lemon juice increased the oropharyngeal swallowing efficiency and safety by significantly reducing the swallowing onset time, pharyngeal delay time (the time required to trigger the pharyngeal swallow), and frequency of aspiration compared with the bolus without lemon juice [49]. However, the high proportion of lemon juice in the bolus was tolerable but not pleasant, according to the patients [49]. Another study mixed lemon juice with liquid barium to assess its effect on swallowing in healthy subjects and in head and neck cancer patients treated with chemoradiation or surgery [144]. Compared with unflavored boluses, the inclusion of lemon juice reduced the pharyngeal transit time (the time required for the bolus to move through the pharynx) in the healthy control subjects as well as in the patients treated for head and neck cancer [144]. This effect was consistent over three evaluation points (at 7–10 days, 1 month, and 3 months) after the patients received cancer treatment [144]. In a different study, the effects on swallowing of cold (4 °C, before delivery) lemon juice diluted in water (10%) was investigated in healthy individuals and patients with a history of stroke [149]. The cold lemon juice shortened the inter-swallow interval in heathy individuals under 60 years of age; however, the velocity (speed) and capacity (volume) of swallowing was reduced [149]. In stroke patients, the velocity of swallowing was also reduced, but the inter-swallow intervals were unaffected [149].

The effects of citric acid on swallowing-related muscle activity and tongue pressure have also been investigated in healthy adults [147,148,152,154,241]. When solutions containing citric acid are consumed, both tongue-palate pressure [148,152,241] and submental muscle activity [147,148,154] are increased in healthy adults. One study reported reduced oral preparation time and increased submental muscle activity when citric acid-containing gelatin cubes were consumed by healthy individuals [154]. Citric acid solutions also improve swallowing safety in patients with oropharyngeal dysphagia [143]. A study conducted in aged nursing home patients with neurogenic oropharyngeal dysphagia reported that swallowing a cold citric acid solution (2.7%) significantly reduced aspiration and penetration compared with water [143].

Citric acid stimulation in peripheral swallowing-related regions has been reported to modify the activity of the cerebral cortex [145,146,242,243,244]. When healthy adults swallow a citric acid solution, blood oxygen level-dependent signals are modified in a range of cortical areas, including the primary somatosensory cortex, anterior cingulate cortex, insula, supplementary motor area, inferior frontal gyrus, and inferior parietal gyrus, as measured by functional magnetic resonance imaging [242]. Furthermore, repeated citric acid swallowing gradually increases the activity in the primary somatosensory cortex and inferior parietal gyrus [242]. Citric acid delivery to the tongue of healthy adults also increases the frequency of swallowing and prolongs hemodynamic responses in cortical swallowing-related areas [145]. During the ingestion of lemon water (lemonade), blood oxygen level-dependent signals in the prefrontal cortex, cingulate gyrus, and sensory/motor cortex are increased in healthy adults [243]. In addition, a study was conducted using the acute application to the tongue of dried filter paper discs with incorporated lemon juice, along with the nasal inhalation of lemon juice odor using a nebulizer (via a nasal canula inserted into both nares) [146]. This treatment led to increased cortical motor evoked potentials from the submental muscles induced by transcranial magnetic stimulation during volitional swallowing in healthy volunteers [146]. These findings suggest that the activation of chemosensory ion channels and taste and odor receptors in the swallowing and olfactory areas can excite cortical swallowing-related neuronal networks during swallowing [146]. In a study of healthy adults, it was reported that the repetitive swallowing of liquid boluses containing citric acid increases corticobulbar excitability [244].

Although the majority of studies have observed improvements in swallowing behavior with the application of ASIC agonists, a few studies have reported opposite effects [149,151,153]. A study in healthy subjects reported that, compared with water, the infusion of acetic acid (10 and 100 mM) solution to the posterior part of the tongue through a tube prolongs the latency to evoke swallowing (as observed by laryngeal movement and the subject’s confirmation) [151]. Other studies have observed reductions in swallowing speed [149,153] and volume [149] when cold (4 °C before delivery to the mouth) lemon juice or citric acid solutions (10%, 50 mL) are consumed by healthy adults [149,153] and patients with a history of stroke [149]. Differences in experimental designs (e.g., the method of detecting the precise timing of swallowing onset [151] or the amount of liquid presented for swallowing [149]) may have influenced the findings of these studies.

## 3. Neurophysiological and Molecular Mechanisms of Improving Swallowing Function via the Activation of Chemosensory Ion Channels by Chemical Stimuli

**Figure 1:**
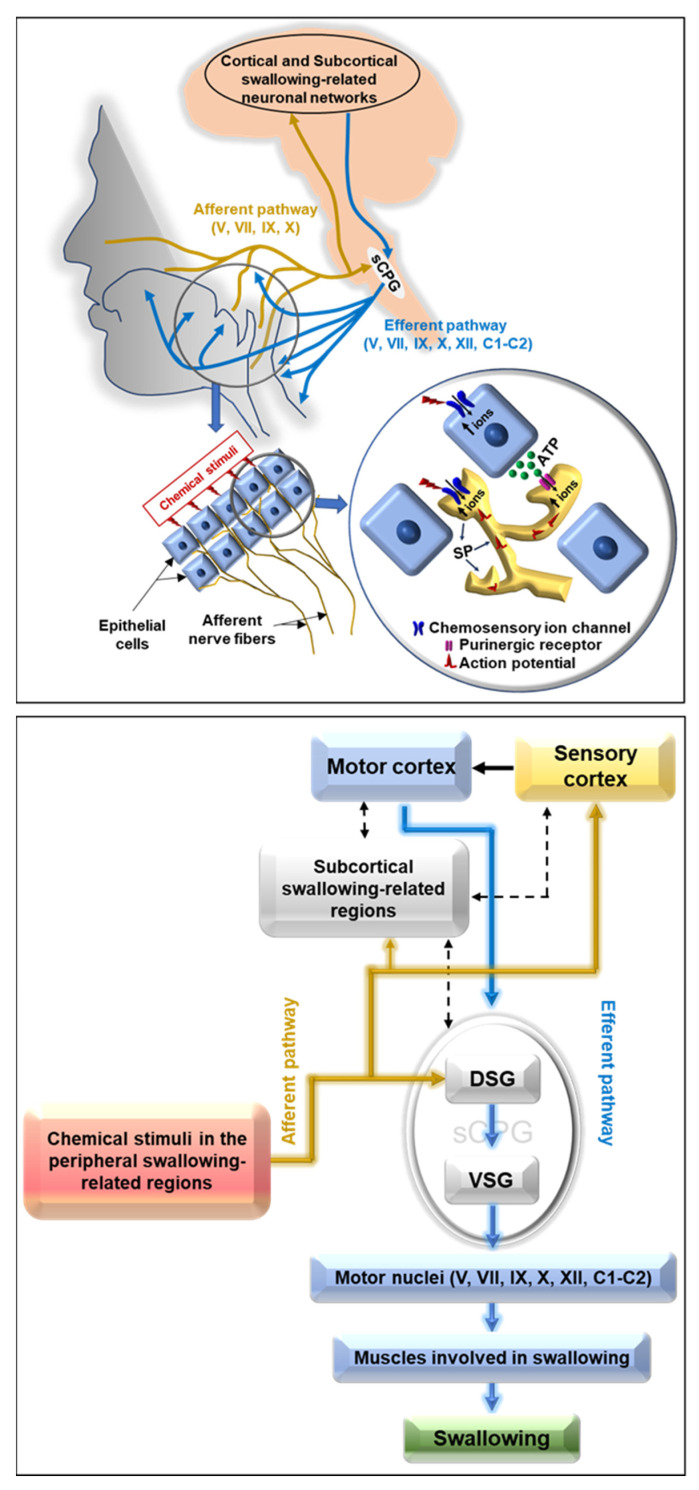
Possible transduction mechanisms and neurophysiological pathways of improving swallowing function via the actions of chemical stimuli applied to peripheral swallowing-related regions. Chemical stimuli applied to peripheral swallowing-related regions can activate chemosensory ion channels expressed in the epithelial cells and nerve fibers in these regions, causing the entry of ions into these structures. The epithelial cells may then release ATP, which can activate purinergic receptors expressed on nearby intra- or sub-epithelial afferent nerve fibers, thus causing the entry of ions into the nerve fibers, leading to the generation of action potentials. Action potentials in the nerve fibers may also be generated by direct ion entry into the nerves through the activation of chemosensory ion channels by chemical stimuli. The action potentials (sensory inputs) then travel via afferent pathways (the V, VII, IX, and X nerves) to the DSG of the sCPG, as well as to the sensory cortex and subcortical swallowing-related regions of the brain. Sensory inputs are then processed by the cortical and subcortical swallowing-related neuronal networks and the sCPG to execute the motor drive for swallowing. The motor output is conveyed to the peripheral swallowing-related muscles through the motor nuclei of the V, VII, IX, X, XII, and C1–C2 nerves. ATP: adenosine triphosphate; DSG: Dorsal swallowing group; SP: Substance P; sCPG: Swallowing central pattern generator; VSG: Ventral swallowing group; V: Trigeminal nerve; VII: Facial nerve; IX: Glossopharyngeal nerve; X: Vagus nerve; XII: Hypoglossal nerve; C1–C2: Cervical nerves 1–2. In the lower part of the figure: Yellow-colored solid lines indicate afferent pathways. Blue-colored solid lines indicate efferent pathways. Black-colored solid line indicates connection between sensory and motor cortex. Black-colored broken lines indicate interconnection among the regions.

The neural mechanisms of swallowing are complex. The pattern of swallowing is generated by a neural network called the swallowing central pattern generator (sCPG), which is located in the brainstem [4,5,16] (Figure 1). It can be divided into two neuronal groups. The dorsal swallowing group (DSG) includes the NTS and contains the generator neurons involved in the triggering, shaping, and timing of the sequential or rhythmic swallowing patterns [4,5,16]. The DSG is activated by sensory inputs from the periphery and by commands from the cerebral cortex [4,5,16]. The ventral swallowing group (VSG) includes the nucleus ambiguus and its adjacent reticular formations and contains switching neurons, which distribute the swallowing drive to the various pools of motor neurons (e.g., trigeminal, facial, glossopharyngeal, vagus, and hypoglossal) involved in swallowing [4,5,16] (Figure 1).

Peripheral sensory inputs are important regulators of swallowing [4,5,65,66,68]. A number of different cranial nerves carry sensory inputs from peripheral swallowing-related regions. The trigeminal nerve (V) carries sensory inputs from the oral cavity and anterior part of the tongue. The facial nerve (VII) carries sensory inputs from the taste buds of the anterior two-thirds of the tongue. The base of the tongue and pharynx are innervated by the glossopharyngeal nerve (IX). In addition, the vagus nerve (X) carries sensory inputs from the laryngopharynx, larynx, and esophagus. Of the swallowing-related regions, the sensory inputs from laryngopharyngeal regions above the vocal cords innervated by the SLN (a branch of the vagus nerve) are reported to be the most potent for evoking the swallowing reflex [245,246]. Electrical stimulation of the SLN can readily elicit this reflex [246,247,248,249]. Information from the periphery travels to the brainstem sCPG as well as to the cerebral cortex to modulate swallowing (Figure 1). An increase in sensory inputs in peripheral swallowing-related regions may reduce the threshold for the sCPG to trigger a swallow response. In an animal study, we observed that the latency to evoke the swallowing reflex was shorter upon the simultaneous electrical stimulation of the SLN and pharyngeal branch of the glossopharyngeal nerve, compared with the independent stimulation of each nerve [248]. Furthermore, the bilateral electrical stimulation of the SLN has been reported to shorten both the latency to trigger swallowing and the inter-swallow intervals compared with unilateral SLN stimulation [249]. In another study, we observed that the number of evoked swallowing reflexes induced by the topical application of TRPV1 or TRPM8 agonists in the laryngopharynx and associated laryngeal regions was markedly reduced after unilateral SLN transection compared with intact bilateral SLNs [116]. These findings suggest that an acute spatiotemporal increase in sensory inputs to peripheral swallowing-related regions may reduce the threshold for the sCPG to trigger the swallow response.

The acute activation of chemosensory ion channels by chemical stimuli applied to peripheral swallowing-related regions may lead to the prolonged release of neurotransmitters in the sCPG. Dense TRPV1 channel localization has been observed in the terminal ends of solitary tract afferents located in the NTS [250,251,252]. The activation of solitary tract afferent nerves generates excitatory postsynaptic currents in the postsynaptic neurons as a result of the release of excitatory neurotransmitters (glutamate) [253,254,255,256,257,258]. The in vitro activation of TRPV1-positive solitary tract afferent nerves leads to both synchronous and long-lasting asynchronous release of glutamate in the NTS, while activation of TRPV1-negative afferents causes only the synchronous release of this neurotransmitter [254,255,256]. Additionally, the amount of asynchronous release of glutamate can be increased by increasing the numbers of activated TRPV1-positive solitary tract afferent nerves [253,255]. The solitary tract contains sensory afferent nerves that innervate peripheral swallowing-related regions, and glutamate is the major excitatory neurotransmitter that triggers the swallowing [4,5,259]. A direct relationship has not yet been established between the swallowing and this prolonged release of glutamate in the NTS by the acute brief activation of TRPV1-positive solitary tract afferent nerves. However, the topical application of a TRPV1 agonist to peripheral swallowing-related regions triggered repeated swallowing reflexes in anesthetized animals in our study, suggesting that a possible relationship exists [116]. In this previous study, a TRPV1 agonist (capsaicin) at an approximately 1000 times lower concentration than a TRPM8 agonist (menthol) evoked a large number of swallowing reflexes when applied to SLN-innervated swallowing-related regions [116]. In human studies, the concentration of TRPV1 agonists needed to improve swallowing function is also lower than that of TRPA1 or TRPM8 agonists [48,118,119,139]. The prolonged asynchronous release of glutamate in the NTS by the activation of TRPV1-positive afferent neurons may be the cause of the greater efficacy of TRPV1 agonists in facilitating swallowing in these human and animal studies. However, there may also be other reasons. A study using biopsy tissues from human oropharyngeal regions reported significantly more TRPV1 mRNA than TRPA1 mRNA [178]. Additionally, TRPV1 immunoreactivity was observed mainly on epithelial cells and subepithelial nerve fibers, whereas TRPA1 immunoreactivity was mainly observed on subepithelial fibroblast-like cells [178]. The presence of a greater number of TRPV1 channels on epithelial cells and sensory nerve fibers may also contribute to the better therapeutic efficacy of TRPV1 agonists. The efficacy of activating sensory nerves can vary even among different agonists of a single chemosensory ion channel. One study reported that the pungency of capsaicin was higher than that of piperine and other TRPV1 agonists (e.g., resiniferatoxin and olvanil) when applied to an animal’s eye (evaluated by the eye wipe test). The pungency was correlated with the lipophilicity of the compounds and their ability to make calcium entry into the sensory neurons [260]. The onset of depolarization by capsaicin in the sensory neurons was also fast, which may help capsaicin generate more action potentials in neurons [260,261,262]. The activation of different subtypes of a channel by different agonists can also be an underlying reason for the variable potency of different agonists [261].

An acute increase in sensory inputs in the peripheral swallowing-related regions can also increase the activity in cortical and subcortical swallowing-related neuronal networks. Various cortical and subcortical areas, including the primary sensorimotor cortex, supplementary motor areas, premotor cortex, anterior cingulate cortex, insula, basal ganglia, and cerebellum, communicate with the brainstem sCPG for the execution of swallowing [263,264,265]. The networks between these areas play an important role in the integration of sensory inputs and motor execution [263,264,265] (Figure 1). In patients with stroke, damage or disruption to swallowing-related neuronal networks in the cortical or subcortical areas leads to difficulties in swallowing [266,267,268], thus indicating the importance of these areas in swallowing. In humans, short trains of electrical stimulation to the pharyngeal region increase blood oxygen level-dependent signals in the sensorimotor cortex [59,71] and increase the excitability of the corticobulbar tracts [269,270]. Additionally, short-term pharyngeal electrical stimulation induces long-term reorganization of the motor cortex in humans [269]. The application of acute chemical stimuli to swallowing-related regions has also been reported to increase activity in cortical and subcortical swallowing-related neuronal networks. During the ingestion of citric acid/lemon water (TRPV1 and ASIC agonists) by healthy adults, there are increased blood oxygen level-dependent signals in cortical areas, including the primary somatosensory cortex, anterior cingulate cortex, insula, supplementary motor area, inferior frontal gyrus, and inferior parietal gyrus, as measured by functional magnetic resonance imaging [242,243]. In addition, cortical motor evoked potentials from the submental muscles during volitional swallowing in healthy adults are increased by a combination of lemon juice stimulation on the tongue and nasal inhalation of lemon juice; this effect persists for at least 90 min following stimuli administration [146]. In patients with oropharyngeal dysphagia associated with aging, stroke, or neurodegenerative diseases, the latency to evoke a cortical response to pharyngeal electrical stimulation is reduced during the acute ingestion of TRPA1 agonists [82]. Together, these findings suggest that acute peripheral chemical stimulation can increase the activity of cortical and subcortical swallowing-related neuronal networks, leading to the facilitation of swallowing function.

Chronic stimulation of peripheral swallowing-related regions by chemical stimuli can also lead to plastic changes (neuronal reorganization) in cortical and subcortical swallowing-related neuronal networks. One study reported an increase in the amplitudes and reduction of the latency to evoke cortical sensorial event-related potentials in response to pharyngeal electrical stimulation in patients chronically ingested nectar boluses containing a TRPV1 agonist [79]. Another study reported that repeated citric acid ingestion in one siting gradually increased activity in the primary somatosensory cortex and inferior parietal gyrus [242]. These findings suggest an improvement in the conduction and integration of sensory information in cortical and subcortical swallowing-related neuronal networks by chronic peripheral chemical stimuli.

Plasticity in synaptic transmission within the NTS (where the sCPG is located) by chronic peripheral chemical stimulation may be possible, although its direct link to swallowing has not yet been elucidated. Studies have reported short- and long-term plasticity in synaptic transmission within the NTS and have related them to lung, airway, and arterial chemoreflexes [271,272]. For example, an animal study reported that chronic exposure to low-oxygen (hypoxic) environments enhances the information transfer between chemosensory afferents and NTS second-order neurons by increasing spontaneous presynaptic neurotransmitter release [273]. A similar kind of plasticity may be possible in NTS swallowing-related networks by chronic peripheral chemical sensory stimulation.

Chemosensory ion channels play integral roles in transducing chemical stimuli to electrical signals in sensory afferent nerves (Figure 1). Chemical stimuli change the ionic permeability of the channels, which can lead to the depolarization of sensory nerves. The expression of various chemosensory ion channels (e.g., TRPs and ASICs) has been observed in peripheral swallowing-related regions, including oral, nasal, pharyngeal, laryngeal, and esophageal regions. These ion channels are mainly expressed on afferent nerve fibers and epithelial cells. The activation of chemo-sensing ion channels can cause the entry of ions (e.g., Ca^2+^, Na^+^) into these structures, leading to their excitation (Figure 1). Because of their superficial localization, epithelial cells are the first cells to be exposed to a stimulus. Studies have reported increases in cations in epithelial cells when they are activated [223,274,275]. Additionally, epithelial cells can communicate with sensory afferents [223,274,275]. Upon activation by chemical stimuli, epithelial cells may release neuroactive molecules (e.g., adenosine triphosphate (ATP)) (Figure 1). Studies suggest that the epithelium, including the nasal, laryngeal, and esophageal epithelium, can release ATP in response to various stimuli, including chemical stimuli [276,277,278,279,280,281,282]. Neuroactive molecules released from epithelial cells can act on the receptors for these molecules (e.g., purinergic receptors) that are expressed on nearby intra- or sub-epithelial afferent nerve fibers [223,274] (Figure 1). Purinergic receptor expression has been observed in intra- and sub-epithelial afferent nerve fibers in laryngopharyngeal and laryngeal regions [283,284]. These receptors are also expressed in the trigeminal, nodose, and petrosal ganglia [285,286,287,288,289]. Thus, afferent nerves can be excited both directly and indirectly (through activation of epithelial cells) by chemical stimuli (Figure 1). Upon excitement, the nerves can release neuropeptides (e.g., substance P and calcitonin gene-related peptide), which may lead to further excitation of the afferent nerves from which they were released, as well as adjacent nerves (Figure 1). Chemical stimuli in peripheral swallowing-related regions may also lead to increased substance P levels in saliva. A randomized controlled trial reported that chronic supplementation of a TRPV1 agonist (capsaicin) in aged patients with oropharyngeal dysphagia increased the salivary substance P levels as well as improving swallowing function [113]. Increased salivary substance P levels are also observed following electrical stimulation of the pharynx [290,291]. Increased substance P levels caused by certain anti-hypertensive drugs (e.g., angiotensin-converting enzyme inhibitors and beta-blockers) have been suggested to improve swallowing functions and reduce the risk of pneumonia [292,293,294,295,296]. Increased substance P levels in peripheral swallowing-related regions can excite the sensory afferent nerves supplying these regions. Studies have reported calcitonin gene-related peptide- or substance P-expressing nerves fibers in peripheral swallowing-related regions, including the tongue, pharynx, epiglottis, and larynx [297,298,299,300,301]. The excitation of afferent nerves by chemical stimuli leads to the generation of action potentials (sensory inputs). These action potentials travel via the sensory branches of different cranial nerves (V, VII, IX, X) that supply peripheral swallowing-related regions, to the sCPG, sensory cortex, and subcortical swallowing-related regions (Figure 1) [4,5,16,39,54]. The sensory inputs are then integrated in the cortical and subcortical swallowing-related neuronal networks and sCPG, to execute the motor drive for swallowing [4,5,16,39,54]. The motor drive then causes sequence of activation and inhibition among more than 25 pairs of muscles involved in swallowing [4,5,16].

The activation of chemosensory ion channels on afferent nerve fibers and epithelial cells is also considered to be responsible for the sensations of irritation, warmth, coolness, and pungency (termed chemesthesis) [223,225,302]. TRPs are highly involved in chemesthesis because they can transduce a wide variety of chemical stimuli [223,225,302]. The trigeminal nerves carry the sensory information to the sCPG and cerebral cortex when a chemical solution is applied to the oral cavity. The projection of trigeminal primary afferents to the NTS has been reported in many studies [303,304,305,306,307]. As the chemical solution passes through the pharyngeal, pharyngolaryngeal, and esophageal regions, the glossopharyngeal and vagus nerves are excited and can carry the sensory information to the sCPG and cerebral cortex [307,308,309,310,311].

Taste receptors are also activated along with chemosensory ion channels upon the application of chemical stimuli to swallowing-related regions. Increased sensory inputs through the nerves that carry taste from swallowing-related regions can excite the sCPG and cortical and subcortical swallowing-related neuronal networks. Taste buds are present in the regions involved in swallowing, including the oral, pharyngeal, and laryngeal regions [312,313,314]. Taste nerves connect to the NTS [312,313,314,315]. The nerves that carry sensation from the laryngopharyngeal regions have been observed to be less responsive to bitter- and sweet-tasting stimuli [316], but more responsive to acids [120,122,316,317]. A range of animal and human studies have reported that sour chemical stimuli (weak acids) facilitate swallowing behavior (Table 1 and Table 2). Sour chemical stimuli have also been found to activate various regions of cortical and subcortical swallowing-related neuronal networks [145,146,242,243,244]. Although the transduction mechanisms of sour taste stimuli in taste receptor cells have not been fully established, various channels, such as epithelial Na^+^ channels [318], hyperpolarization-activated cyclic nucleotide-gated channels [319], ASICs [236], polycystic-kidney disease-like (PKD) ion channels (PKD2L1 and PKD1L3) [320], resting K^+^ channels, Kir2.1 [321], proton-selective ion channel (otopetrin 1) [322] have been implicated in sour taste detection [314].

The inhalation of volatile chemical compounds (e.g., black pepper oil, vanillin) has also been observed to facilitate swallowing function [140,142]. Volatile chemical compounds may activate chemosensory ion channels present in the epithelial cells and nerve fibers of the nasal cavity, which may lead to the excitation of branches of the trigeminal nerves that supply the nasal cavity [159]. Volatile chemical compounds may also be released during the chewing of different kind of foods, and may pass to the nasal cavity through the retronasal pathway. Olfactory receptors may also play a role in facilitating swallowing. Increased sensory inputs through the olfactory nerves upon excitation by odor molecules may lead to an increase in the excitability of cortical and subcortical swallowing-related neuronal networks [140]. One study has reported increased insular cortex activity following nasal inhalation of black pepper oil, which was associated with a facilitation of triggering of the swallowing [140]. Nasal inhalation of black pepper oil may also directly activate TRPs present in the nasal, pharyngeal, and laryngeal regions.

Carbonated liquid or boluses have been reported to influence swallowing function in healthy adults and patients with oropharyngeal dysphagia [244,323,324,325,326,327,328]. Chemosensory ion channels can be activated by carbonated liquids that contain dissolved CO_2_. Carbonated drinks elicit a sensation of tingling and irritation when applied to the oral cavity [329]. Although there may also be some contributions by mechanosensitive channels (activated by the bursting of tiny CO_2_ bubbles) [330,331], recent studies have reported that the sensation elicited by CO_2_ is primarily of chemogenic origin [223,302,329,332,333,334]. During the ingestion of carbonated liquids, CO_2_ and water can be converted into carbonic acid by the action of carbonic anhydrase enzymes [302,333,334]. In mammals, various isoforms of this enzyme are observed in the cell membranes and cytosol of cells [335]. The converted carbonic acid can activate ASICs, TRPV1, or other acid-activating channels. One study observed that many CO_2_-sensitive afferent neurons from the cat cornea are also sensitive to a TRPV1 agonist (capsaicin); however, their activation is not blocked by a TRPV1 antagonist (capsazepine), suggesting a TRPV1-independent mechanism [336]. Another study reported that the sensations evoked by carbonated water on the human tongue are partially inhibited by TRPV1 desensitization, suggesting the partial involvement of TRPV1 [332]. In the rat esophagus, CO_2_ perfusion-induced hyperemia can be inhibited by a TRPV1 antagonist (capsazepine), thus supporting a TRPV1-mediated action [238]. CO_2_ is lipid soluble, and can therefore easily pass through cell membranes. One study suggested that CO_2_ is converted to carbonic acid intracellularly, where acidification subsequently activates TRPA1 [337]. This study observed that a subpopulation of trigeminal neurons that express TRPA1 are activated by CO_2_. Additionally, CO_2_ activates TRPA1 channels, but not TRPV1 channels, that are heterologously expressed in human embryonic kidney 293 cells [337]. In addition to inducing sensations of tingling and irritation, carbonated liquids also elicit a sour taste sensation [338]. Carbonated liquids are reported to activate taste receptor cells that express the heteromeric PKD ion channels PKD2L1 and PKD1L3 (members of the TRPP family) in mammals [320]. Chorda tympani nerve responses to CO_2_ and citric acid exposure are abolished in genetically engineered mice in which neurotransmitter release from PKD2L1-expressing taste receptor cells is blocked [338]. Carbonic anhydrase enzymes may also play a role in the sour taste detection of carbonated liquids [338,339]. Expression of carbonic anhydrase 4 (an isoform of this enzyme) has been observed on the extracellular surface of type III sour-sensing taste receptor cells that co-express PKD2L1 [338,339], suggesting that this enzyme is involved in extracellular acidification. Carbonated liquids may also activate other acid-sensing ion channels, such as ASICs and acid-sensitive K^+^ channels [238,340,341]. In the rat esophagus, CO_2_ solution-induced hyperemia can also be inhibited by an ASIC antagonist (amiloride), suggesting the activation of ASICs [238]. Another study observed the involvement of tandem P-domain K^+^ channel 1 in increasing chemoafferent discharge from the carotid sinus nerves caused by increased blood CO_2_ levels in mice [341]. In addition, the activation of ASIC1a in the amygdala, caused by reduced pH arising from increased CO_2_ inhalation in mice, has also been observed [340]. These findings suggest the possible involvement of various chemosensory ion channels in the influence of carbonated liquids or boluses on swallowing function, although no direct link has yet been established.

The unmyelinated nerves of swallowing-related regions may be more activated by the application of chemical stimuli. In our animal studies, we observed that TRPV1, TRPM8, and ASIC3 are largely expressed in unmyelinated afferent nerves from the SLN-innervating swallowing-related regions [116]. The influence of unmyelinated nerves on facilitating the triggering of the swallowing is of particular interest. These nerves can be utilized to improve swallowing function in older patients with oropharyngeal dysphagia, because it has been observed in human studies that the number of myelinated nerves in the SLN are gradually reduced in the aging process [74,75].

## 4. Conclusions

Evidence from various studies suggests that the activation of chemosensory ion channels in peripheral swallowing-related regions may be a potential strategy for the development of new active pharmacological treatments of oropharyngeal dysphagia. The advantages of this strategy are that it does not require specific costly equipment and is relatively cheap and easy to conduct, and patient compliance may also be good. Patients are not required to swallow tablets or capsules; rather, the channel agonists can be mixed with ingestible boluses. Because patients with oropharyngeal dysphagia often face difficulties in swallowing tablets or capsules [36,342], this advantage may provide added benefits in terms of patient compliance. In a considerable number of human studies, low concentrations of natural agonists of some TRPs (e.g., capsaicin and piperine) have been mixed with ingestible boluses to improve swallowing functions (Table 2). These natural agonists are phytochemicals found in culinary herbs and spices, and are advantageous because they may not have serious side effects at low concentrations. Many phytochemicals and active compounds of various botanicals can activate TRPs [161], and therefore have the potential to facilitate swallowing. In future studies, phytochemicals of various botanicals should be investigated in animal and human trials to investigate their potency, specificity, and dose of action to improve swallowing functions. The TRP family has many members, but only TRPV1, TRPA1, and TRPM8 channels have so far been targeted in studies of dysphagia management. The expression of other TRPs (e.g., TRPV2, TRPV4, and TRPM3) has been reported in swallowing-related regions and ganglia [167,343,344,345]. Thus, the functional roles of these TRPs in swallowing processes need to be investigated in future research, as well as whether they can be targeted for dysphagia management. Along with TRPs, other chemosensory ion channels (e.g., ASICs and purinergic channels) can also be targeted. Highly potent synthetic agonists of these channels can be considered in basic research; however, their safety needs to be assured before they can be used in clinical trials.

To date, several mid-term clinical trials have provided evidence of the development of neuroplasticity in swallowing-related neuronal networks following chronic supplementation of some chemosensory ion channel agonists. These trials suggest that both short- and long-term therapeutic benefits can be achieved using this strategy [79,114,119,136,137]. Chronic agonist supplementation is well tolerated by patients and no adverse events related to the agonists have been reported [79,114,137,139]. However, currently, the effect of long-term supplementation is unknown. Therefore, whether efficacy is retained in long-term agonist supplementation, and the possible development of adaptation or desensitization, needs to be studied in long-term randomized, controlled, multi-center trials of large numbers of patients with oropharyngeal dysphagia. Understanding the maintenance capability of neuroplasticity over time with short- or mid-term supplementation is also important. Furthermore, patient phenotype is another important issue to be considered. The etiology of oropharyngeal dysphagia and its accompanying health conditions can vary among patients; therefore, same treatment strategy may not be effective for every patient phenotype [41,54,114]. Although patient recruitment may be challenging, clinical trials with large numbers of patients with the same phenotypes need to be conducted, to understand the effectiveness of different treatment strategies within the same patient phenotype. Studies combining the peripheral chemosensory ion channel activation strategy with other promising treatment strategies (e.g., cortical neurostimulation or pharyngeal electrical stimulation) may also need to be conducted.

In summary, oropharyngeal dysphagia treatment strategies are gradually changing from compensatory strategies toward strategies that promote the recovery of normal swallowing physiology and provide neuroplasticity in swallowing-related neuronal networks. Targeting chemosensory ion channels in peripheral swallowing-related regions may be a promising pharmacological treatment strategy for the future management of oropharyngeal dysphagia.
